# First Record of *Hepatospora eriocheir* Infection in the Chinese Mitten Crab *Eriocheir sinensis* in the Baltic Sea

**DOI:** 10.3390/pathogens15070681

**Published:** 2026-06-26

**Authors:** Magdalena Stachnik, Monika Normant-Saremba, Anna Kycko

**Affiliations:** 1 Department of Parasitology and Invasive Diseases, Bee Diseases and Aquatic Animal Diseases, National Veterinary Research Institute, Al. Partyzantów 57, 24-100 Puławy, Poland; 2 Faculty of Oceanography and Geography, Department of Marine Ecology, University of Gdańsk, Al. M. Piłsudskiego 46, 81-378 Gdynia, Poland; monika.normant@ug.edu.pl; 3 Department of Research Support, National Veterinary Research Institute, Al. Partyzantów 57, 24-100 Puławy, Poland; anna.kycko@piwet.pulawy.pl

**Keywords:** crustacean parasite, shellfish pathogen, wild and aquaculture-relevant host, microsporidium, host–parasite interaction, molecular diagnosis, Baltic Sea, invasive aquatic host

## Abstract

*Hepatospora eriocheir* is a microsporidian parasite of the Chinese mitten crab *Eriocheir sinensis*, an invasive decapod now widely distributed in European inland and coastal waters. Although the host is common across much of Europe, confirmed European records of *H. eriocheir* have remained scarce and, until now, have not included the Baltic Sea region. In this study, 15 adult *E. sinensis* collected from the Vistula Lagoon, southern Baltic Sea, were examined using gross pathological assessment, wet-mount microscopy, histopathology, PCR amplification, Sanger sequencing, and phylogenetic analyses of parasite SSU rRNA and host COI sequences. Hepatopancreatic alterations were observed in several individuals, ranging from pale discolouration and friability to loss of normal tissue organisation. Spores were detected in fresh squash preparations from affected tissue, and histology revealed epithelial disruption, intratubular spore accumulation, and necrotic changes consistent with progressive microsporidian infection. Molecular screening confirmed *H. eriocheir* in 60.0% of crabs, with positive cases occurring in both females and males. The parasite sequences formed a single, well-supported clade and were highly similar to previously reported *H. eriocheir* sequences from the United Kingdom and China. Host COI sequences represented three mitochondrial haplotypes, indicating that the infection occurred across more than one host mitochondrial haplotype background. These findings constitute the first record of *H. eriocheir* in *E. sinensis* from the Baltic Sea and support the hypothesis that infected crabs reaching the Vistula Lagoon are connected with the wider North Sea invasion system rather than an isolated Baltic lineage.

## 1. Introduction

Microsporidia are obligate intracellular, spore-forming eukaryotic parasites with a broad host range and substantial medical, veterinary, and ecological relevance. In aquatic systems, microsporidia infect hosts ranging from protists to vertebrates, but crustaceans are particularly important hosts because infections may remain externally inapparent while causing severe internal pathology. Their importance in aquatic animal health has increased with the intensification of aquaculture, translocation of live organisms, and movement of invasive hosts across hydrographic barriers [1,2]. Among decapod-associated microsporidia, *Hepatospora eriocheir* is notable because it targets the hepatopancreatic epithelium of the Chinese mitten crab *Eriocheir sinensis*. The parasite was first described from Asian mitten crabs as *Endoreticulatus eriocheir* and was later reassigned to *Hepatospora* after combined morphological and phylogenetic analysis of material from the Thames Estuary, United Kingdom [3,4]. Subsequent work from China linked *H. eriocheir* infection with hepatopancreatic necrosis disease in pond-reared *E. sinensis*, and metabolomic studies indicate that infection may disrupt energy and intermediary metabolism [5,6]. These findings are consistent with the central physiological role of the hepatopancreas in digestion, nutrient storage, detoxification, and endocrine regulation in decapod crustaceans. The host, *E. sinensis*, occupies a dual biological and socioeconomic status. It is a valued aquaculture species in East Asia, but in Europe it is one of the most prominent invasive aquatic crustaceans. The species has been included among globally significant invasive alien species and has spread across European rivers, estuaries, and coastal waters since the early twentieth century [7,8,9,10]. Its catadromous life cycle, broad salinity tolerance, high mobility, and association with shipping and riverine networks make it an efficient vector not only of its own dispersal but also of associated symbionts and pathogens. In addition to microsporidian infections, the Chinese mitten crab has been associated with a range of other pathogens and disease conditions reported from this host or from decapod crabs more broadly, including bacterial, viral, fungal, oomycete, and protistan agents [11]. This broader pathogen spectrum is relevant in the context of biological invasions, because invasive crab populations may act as carriers of associated infectious agents and may contribute to pathogen movement between geographically separated aquatic systems [12,13].

The Baltic Sea is a particularly interesting region for understanding this host–parasite system. Mitten crabs have been recorded from Polish waters for decades, including the Gulf of Gdansk, the Vistula Lagoon, and adjacent coastal areas [14,15]. However, low salinity in much of the southern and eastern Baltic limits successful completion of the host life cycle. Consequently, Baltic occurrences are widely interpreted as at least partly dependent on recurrent influx from well-established donor populations, especially those associated with the North Sea and Elbe system [15,16]. Polish records are therefore epidemiologically relevant because they may represent repeated arrivals of crabs and their parasites into a brackish habitat. Despite the broad distribution of *E. sinensis* in Europe, confirmed detections of *H. eriocheir* outside Asia have been limited. The Thames Estuary records demonstrated that the parasite had reached the invaded European range [4,12], but the extent of its distribution within the North Sea to Baltic invasion corridor remains poorly resolved. This gap is important because externally healthy crabs may harbour advanced hepatopancreatic infection, and routine field monitoring rarely includes histology and molecular parasite screening.

During dissection of adult *E. sinensis* collected from the Vistula Lagoon, conspicuous differences in hepatopancreas colour, firmness, and tissue integrity were observed. These changes resembled the gross alterations previously associated with *H. eriocheir* infection. The present study therefore aimed to determine whether *H. eriocheir* occurs in Baltic *E. sinensis*, to characterize the associated pathology, and to place both host and parasite sequences into a phylogenetic and biogeographic framework. The work integrates gross pathology, microscopy, histopathology, PCR, Sanger sequencing, and maximum likelihood phylogenetic analysis to assess whether the Baltic record fits a wider North Sea-linked invasion pattern.

## 2. Materials and Methods

### 2.1. Sample Collection and Gross Examination

Fifteen adult specimens of *Eriocheir sinensis* were collected in October 2020 from the south-eastern part of the Vistula Lagoon, between Tolkmicko (54.32056° N, 19.53056° E) and Frombork (54.35000° N, 19.68300° E), in the southern Baltic Sea. Crabs were obtained during commercial fishing using fyke nets. At the time of sampling, water temperature was 9 °C and salinity was 3 PSU, which reflects the brackish conditions characteristic of this lagoonal habitat. In the laboratory, each crab was examined externally and dissected using a standardized procedure. Sex was determined from abdominal morphology, and carapace width was measured to the nearest 0.1 mm. Before dissection, crabs were chilled on ice for 1 h to reduce handling movement and tissue disruption. After removal of the dorsal carapace, the hepatopancreas was assessed macroscopically for colour, apparent size and development, firmness, tissue cohesion, and preservation of the tubular structure. Hepatopancreata were classified as grossly unaltered when they were well developed, compact, yellow to yellow-orange, and showed preserved tubular architecture. Tissues were considered altered when they showed pallor or grey-white discolouration, reduced firmness, friability, loss of tissue cohesion or reduced apparent volume. This assessment was descriptive and was not used alone to determine infection status. Hepatopancreatic tissue and walking leg muscle were collected separately from each individual and preserved in 96% ethanol for molecular analysis.

### 2.2. Wet-Mount Examination and Histopathology

Fresh hepatopancreatic tissue from three individuals selected to represent clearly different gross hepatopancreatic appearance was examined immediately after dissection. Small fragments of tissue, approximately 10 mm^3^, were placed on glass slides in a drop of water, gently squashed under a coverslip, and examined by light microscopy at 100–400× magnification. The aim of wet-mount examination was to detect spores or other structures indicative of microsporidian infection in unfixed material and to compare these results with the results of PCR and histological examinations. Two hepatopancreatic sections from each of three selected individuals, representing both grossly unaltered and altered appearances, were fixed in 10% neutral-buffered formalin, processed routinely, embedded in paraffin, sectioned at 5 µm, and stained with haematoxylin and eosin according to a standard protocol [17]. Sections were examined using an Axiolab 5 microscope (Carl Zeiss, Oberkochen, Germany). Representative micrographs were acquired using a Carl Zeiss Axiocam 208 digital camera (Carl Zeiss, Oberkochen, Germany). Histological evaluation focused on tubular architecture, epithelial integrity, localisation and density of spores, luminal obstruction, cellular degeneration, and necrosis.

### 2.3. DNA Extraction, PCR Amplification, Sequencing and Phylogenetic Analysis

Genomic DNA was extracted from hepatopancreatic tissue and walking leg muscle using the QIAamp DNA Mini Kit (Qiagen, Hilden, Germany), according to the manufacturer’s protocol. Two genetic markers were targeted. A partial SSU rRNA fragment of the microsporidian parasite was amplified from hepatopancreatic DNA using the MF1/MR1 primer pair described for microsporidian detection [18]. Host mitochondrial COI was amplified from muscle DNA using the universal LCO1490/HCO2198 primer pair [19]. PCR products were sequenced commercially by Genomed (Warsaw, Poland). Forward and reverse chromatograms were inspected manually, assembled into consensus sequences, and trimmed to remove low-quality terminal regions. Only sequences with unambiguous chromatograms and sufficient length for comparative analysis were retained. Sequence editing and assembly were performed in Geneious R7.1.9 [20]. Host COI and parasite SSU rRNA sequences were aligned separately using MUSCLE [21], as implemented in MEGA X [22]. Alignments were checked manually, and sequence ends were trimmed to homologous positions shared by the study sequences and reference material. Phylogenetic analyses were conducted separately for host and parasite datasets. Representative GenBank sequences were selected to cover relevant *Eriocheir* COI diversity and microsporidian taxa related to *Hepatospora*. Maximum likelihood (ML) trees were inferred in MEGA X under the Tamura-Nei substitution model [23], with a discrete Gamma distribution to account for among-site rate heterogeneity. Initial trees for heuristic searches were generated automatically using Neighbor-Joining and BioNJ procedures and then optimised under maximum likelihood. Nodal support was assessed with 1000 bootstrap replicates. Bootstrap values of 70% or greater were interpreted as moderate support, and values above 80% were treated as strong support.

### 2.4. Statistical and Integrative Analysis

The observed frequency of the parasite within the sample was calculated as the proportion of PCR-positive crabs among all examined individuals. Infection frequency was compared between males and females using Fisher’s exact test. Carapace width was compared between PCR-positive and PCR-negative crabs using Welch’s *t*-test because of the groups were small and unequal in size. A threshold of *p* < 0.05 was used for statistical significance. For each individual, gross findings, wet-mount observations, histopathology, PCR results, and sequence data were interpreted jointly. This integrative approach was used because visible hepatopancreatic alteration alone cannot confirm infection status or aetiology, whereas molecular detection alone does not describe tissue damage or pathological significance.

## 3. Results

### 3.1. Gross Morphology, Wet-Mount Examination, and Histopathology

**Figure 1:**
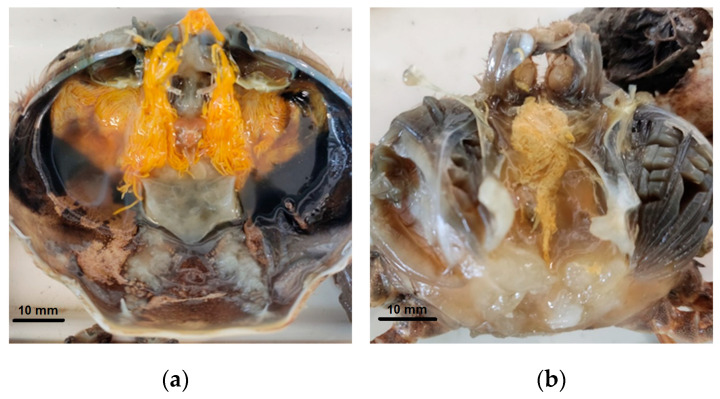
Comparison of hepatopancreas morphology in *Eriocheir sinensis*. (**a**) individual no. 1: Normal-appearing, compact yellow hepatopancreas with preserved gross structure; positive for *Hepatospora eriocheir* by wet-mount examination and PCR. (**b**) individual no. 7: Altered hepatopancreas, pale and structurally degraded tissue; positive for *H. eriocheir* by wet-mount examination and PCR. Photo credit: M. Stachnik.

The sample consisted of 15 adult *E. sinensis*, including eight males and seven females. No consistent external signs of disease were observed before dissection. In contrast, internal examination revealed marked variation in hepatopancreas appearance. In apparently unaffected crabs, the hepatopancreas was well developed, compact, and intensely yellow to yellow-orange (Figure 1a). Affected individuals showed a paler organ, reduced firmness, friable consistency, and, in several cases, grey-white or almost white areas (Figure 1b). The most severely altered hepatopancreata were visibly smaller and showed structural degradation, whereas most PCR-negative individuals had compact yellow to yellow-orange organs. However, macroscopic appearance was not fully concordant with molecular results because some PCR-positive individuals retained a yellow hepatopancreas. Therefore, gross appearance should not be used as the sole criterion for determining infection status.

**Figure 2:**
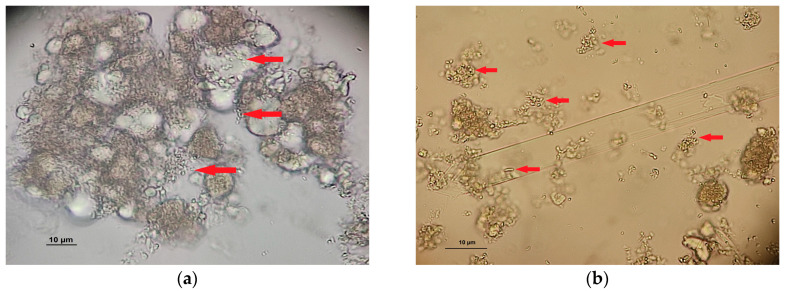
Wet-mount detection of microsporidian spores in the hepatopancreas of *E. sinensis*. Fresh squash preparations of hepatopancreatic tissue from individual no. 1 (**a**) and no. 7 (**b**), showing disrupted tissue architecture and numerous ovoid, refractile spore-like bodies indicated by red arrows. Original magnification: 400×. Photo credit: M. Stachnik.

**Figure 3:**
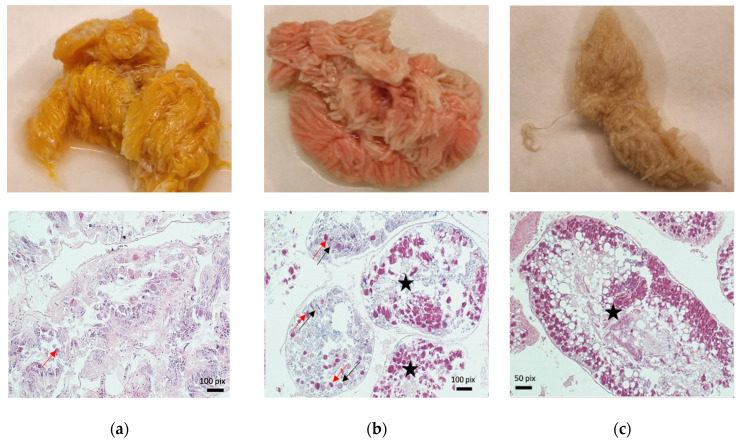
Gross and histopathological progression of hepatopancreatic lesions in *Eriocheir sinensis* infected with *Hepatospora eriocheir.* (**a**–**c**) Macroscopic appearance of hepatopancreatic tissue showing progressive change from compact yellow-orange tissue to pale, friable, and necrotic tissue (upper row). Corresponding histopathological changes showing focal epithelial infection, progressive disruption of hepatopancreatic tubules, accumulation of parasite spores within tubular lumina, and extensive necrosis with loss of normal tissue architecture. Red arrows indicate altered epithelial cells and eosinophilic nuclear changes; black arrows indicate basophilic inclusions; black stars mark tubule lumina densely filled with parasite spores (lower row). Scale bars as indicated. Images: M. Stachnik.

Wet-mount preparations from individuals nos. 1, 2 and 7, selected to represent different gross hepatopancreatic appearances, revealed variable numbers of spores consistent in microsporidia infection (Figure 2). This direct observation supported a link between macroscopic lesions and the presence of a spore-forming parasite and justified histological assessment of tissue-level damage (Figure 3a–c). Because only three purposively selected individuals were examined by wet-mount, these observations were not used to estimate the frequency of infection in the sample.

Histopathology confirmed hepatopancreatic lesions consistent with active microsporidian infection (Figure 3a–c). In mild lesions, the tubular organization remained partly preserved, but focal epithelial infection and scattered spores were present (Figure 3a). In more advanced lesions, hepatopancreatic tubules were distended or disrupted, epithelial cells were degenerated or detached, and tubular lumina contained abundant spores (Figure 3b). In severe lesions, normal hepatopancreatic architecture was extensively replaced by parasite-rich material, cellular debris, and necrotic tissue (Figure 3c).

The principal microscopic features were epithelial disorganization, intratubular spore accumulation, cellular degeneration, eosinophilic nuclear alteration, and basophilic inclusion-like parasite stages within hepatopancreatic epithelial cells (Figure 3a–c, lower row). The distribution and character of the lesions were not consistent with post-mortem autolysis alone. The concordance of gross lesions, spores in fresh preparations, and tissue-associated parasite stages supports a diagnosis of biologically significant hepatopancreatic microsporidiosis in the affected individuals.

### 3.2. Molecular Detection of Parasite and Host Data

Because of the small sample size, statistical analyses were considered exploratory. The observed frequency of PCR positivity between sexes was compared using Fisher’s exact test. Carapace width was compared between PCR-positive and PCR-negative individuals using Welch’s *t*-test. Statistical results were not used to infer population-level relationships.

Five of eight males and four of seven females were PCR-positive. Fisher’s exact test provided no evidence of a sex-associated difference within this small sample (*p* = 1.00). Mean carapace width was 67.8 mm (SD 4.4) in PCR-positive crabs and 62.6 mm (SD 7.6) in PCR-negative crabs. The exploratory comparison did not provide evidence of a difference within the examined sample (Welch’s *t*-test, *p* = 0.17). These analyses had low statistical power and should not be interpreted as demonstrating the absence of biological associations. Molecular findings were broadly consistent with gross and histological observations. Crabs with pale, friable, or white hepatopancreatic tissue were usually PCR-positive, whereas individuals with compact, yellow-orange hepatopancreas were generally PCR-negative. However, PCR positivity was also recorded in some crabs whose hepatopancreas appeared yellow macroscopically, indicating that early or less severe infection may not always be recognized by gross examination alone (Table 1).

### 3.3. Host COI Diversity, Population Context, and Parasite Phylogeny

**Figure 4:**
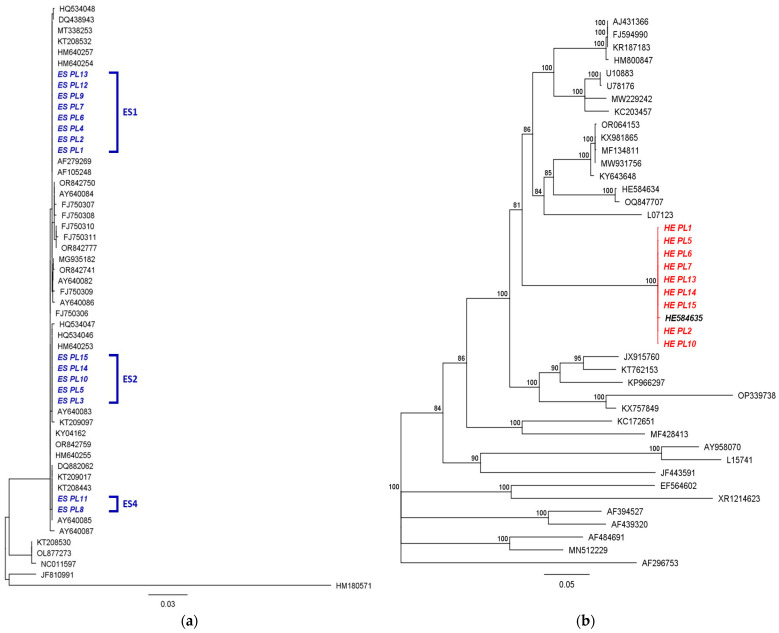
Maximum likelihood phylogenies of Chinese mitten crab *Eriocheir sinensis* and *Hepatospora eriocheir*. (**a**) Evolutionary analysis of cytochrome c oxidase subunit I (COI) sequences of *E. sinensis* from the Baltic Sea, including Polish haplotypes ES1, ES2, and ES4 marked in blue, together with related sequences obtained from GenBank. (**b**) Evolutionary analysis of SSU rRNA sequences of *H. eriocheir* obtained from the analysed *E. sinensis*, marked in red, together with related *Hepatospora* spp. sequences from GenBank.

Host COI analysis revealed three mitochondrial haplotypes among the analysed crabs, designated ES1, ES2, and ES4 (Figure 4a). Thus, the Vistula Lagoon sample was not genetically uniform at the mitochondrial level, even though all individuals were collected from one lagoonal region. PCR-positive crabs were represented in more than one host haplotype, indicating that infection was not restricted to a single maternal lineage in the analysed material.

In contrast, the parasite SSU rRNA dataset showed little sequence variation. All *H. eriocheir* sequences obtained in this study clustered in a single, strongly supported clade (Figure 4b). Internal branch lengths among Baltic sequences were short, and no distinct Baltic sub-lineage was evident. The sequences grouped closely with published *H. eriocheir* sequences from the Thames Estuary and China, supporting identification as *H. eriocheir* within its microsporidian lineage. The combined host and parasite results therefore show an asymmetric pattern: several host mitochondrial haplotypes occurred in the sample, whereas the parasite lineage was genetically highly conserved across infected crabs. This pattern is compatible with host-mediated introduction or circulation of a single dominant parasite lineage within a genetically heterogeneous invasive host population.

Gross examination, wet-mount microscopy, histopathology, and molecular analysis gave mutually supportive evidence of *H. eriocheir* infection in Baltic *E. sinensis*. Pale and structurally degraded hepatopancreatic tissue was associated with spores in fresh preparations, histological lesions consistent with microsporidian development, and PCR confirmation of *H. eriocheir*. The infection was detected in both sexes and across more than one host mitochondrial background. The parasite phylogeny did not indicate independent emergence of a divergent Baltic lineage. Instead, the Baltic sequences clustered closely with previously confirmed *H. eriocheir* isolates, while host COI data were consistent with the broader mitochondrial diversity already described from European mitten crabs. When interpreted alongside published evidence that Polish non-self-sustaining populations are connected with Elbe and North Sea source systems, the Baltic record is most consistent with the arrival of infected crabs via the regional invasion network rather than an isolated local parasite cycle.

## 4. Discussion

This study documents the first confirmed record of *Hepatospora eriocheir* in *Eriocheir sinensis* from the Baltic Sea. Together with the earlier British records, the finding extends the known European distribution of this microsporidian beyond the British Isles and indicates that *H. eriocheir* may be underdetected in invaded European decapod populations [4,12]. The relatively high proportion of PCR-positive crabs in the present sample should be interpreted cautiously because only 15 individuals were examined. Nevertheless, the convergence of gross, microscopic, histological, and molecular evidence makes the diagnostic conclusion robust.

The hepatopancreatic lesions observed here are biologically meaningful. The hepatopancreas is one of the principal organs controlling digestion, absorption, storage, detoxification, and intermediary metabolism in decapods. Progressive epithelial disruption, intratubular spore accumulation, and replacement of functional tissue by necrotic and parasite-filled material are therefore unlikely to be neutral for the host. Although physiological performance was not measured, the lesions are compatible with impaired digestive and metabolic capacity. This interpretation is consistent with previous reports linking *H. eriocheir* with hepatopancreatic necrosis disease and altered metabolic profiles in Chinese mitten crabs [5,6]. A key diagnostic point is that macroscopic examination alone would probably underestimate infection frequency. Several strongly altered hepatopancreata were visibly suspicious, but PCR positivity also occurred in crabs whose hepatopancreas remained yellow at gross inspection. This suggests that early or moderate infections may be missed unless molecular screening and/or histology are included. For invasive decapods, where field surveillance often focuses on distribution records rather than pathology, such cryptic infections can remain invisible for long periods. Although the present study focused on *H. eriocheir*, the pathological significance of invasive mitten crab should be considered in a broader context of pathogen carriage. No lesions or parasite stages morphologically suggestive of other readily recognisable systemic infections were observed in the examined hepatopancreatic sections. Nevertheless, histological examination was restricted to hepatopancreatic tissue from three individuals and pathogen-specific molecular tests were performed only for *H. eriocheir.* Therefore, the present study cannot exclude the occurrence of other pathogens in the analysed crabs. Broader surveillance, including targeted molecular assays and examination of additional tissues, would be required to assess the full pathogen spectrum carried by *E. sinensis* in the Baltic region.

The biogeographic interpretation requires integration of the host population context and parasite phylogeny. Low salinity restricts successful reproduction of *E. sinensis* across large areas of the southern and eastern Baltic, and Polish records are commonly considered to involve recurrent immigration rather than fully independent local recruitment [14,15,16]. The host COI data obtained here do not support an isolated Baltic lineage. Instead, the presence of multiple haplotypes and previous evidence of genetic connectedness between Polish non-self-sustaining populations and the Elbe system support the placement of the Vistula Lagoon material in the wider North Sea-linked invasion network [16]. Within this framework, secondary arrival of infected crabs from established North Sea donor systems is the most parsimonious explanation for the Baltic occurrence of *H. eriocheir*. German waters and the Elbe catchment are plausible contributors because they represent nearby self-sustaining components of the regional invasion system. However, the present data do not allow precise attribution to a single donor region. Historic ship-mediated transport, repeated secondary dispersal, and movement through connected estuarine and coastal systems may all have contributed to the broader European host distribution [8,9,10].

The parasite phylogeny is consistent with this interpretation but does not resolve the exact donor area. Baltic *H. eriocheir* sequences formed a conserved clade closely related to sequences from the Thames Estuary and China [4,5]. This pattern is more consistent with movement of an already established parasite lineage through invaded host populations than with independent emergence of a distinct Baltic parasite. The host–parasite asymmetry observed here, namely several host COI haplotypes but little parasite SSU rRNA variation, is compatible with co-introduction and subsequent spread of a comparatively homogeneous parasite lineage within a genetically mixed invasive host population. Such patterns are expected when repeated host introductions maintain mitochondrial diversity while parasite diversity is reduced by founder effects, host filtering, or the relatively conserved nature of the SSU rRNA marker used [24,25].

Microsporidian emergence associated with host movement, trade, or range expansion is documented in several host–parasite systems. *Nosema ceranae*, originally associated with the Asian honey bee, has become an important emergent microsporidian in European honey bees [26]. *Enterocytozoon bieneusi* is another example of a microsporidian with broad host associations and substantial veterinary and public health relevance [27]. In crustaceans, hepatopancreatic microsporidia such as *Enterocytozoon hepatopenaei* illustrate how tissue-specific infections can become major disease constraints in aquatic hosts [18]. These examples do not imply identical transmission ecology for *H. eriocheir*, but they show that microsporidia can spread effectively across host populations and remain epidemiologically important even when clinical signs are not externally obvious. The ecological consequences of *H. eriocheir* infection in the Baltic Sea remain unresolved. Infection could reduce individual crab condition, alter energetic allocation, impair migration, or lower reproductive output. Such effects might theoretically reduce the invasive potential of *E. sinensis* in sink regions, but this should not be assumed without functional data. A parasite that harms an invasive host may also maintain infection reservoirs, alter predator-prey interactions, or create opportunities for spillover if related native crustaceans are susceptible. At present, spillover from *E. sinensis* to native Baltic decapods has not been demonstrated, and the host range of the parasite in this region requires targeted testing.

The present study has several limitations that should guide future work. The sample size was modest, sampling was restricted to a single period and one lagoonal area, and phylogeographic inference was based primarily on COI and SSU rRNA markers. These markers are appropriate for parasite detection and initial phylogenetic placement, but they do not provide sufficient resolution to reconstruct fine-scale transmission routes. Broader sampling from German, Danish, Dutch, Belgian, British, and additional Baltic localities or other decapod species would be required to determine whether *H. eriocheir* is broadly established along the invasion corridor between the North Sea and the Baltic Sea or reflects repeated, localised introductions. Bateman et al. [12] demonstrated that a concatenated multigene phylogeny provides improved resolution for assessing relationships among *Hepatospora* spp. isolates from different decapod hosts. Comparable analysis of Baltic material would be required to assess fine-scale variation, host-associated variants, and possible transmission routes. Despite these limitations, the integrated morphological, histopathological, and molecular evidence supports the conclusion that *H. eriocheir* is now part of the documented pathogen fauna of invasive *E. sinensis* in the Baltic Sea. This finding has practical relevance for invasive species management, aquatic animal health surveillance, and crustacean pathology. It also demonstrates that targeted examination of internal organs, particularly using molecular methods, can reveal parasite introductions that may remain undetected if assessment is based solely on external inspection or gross lesions.

## 5. Conclusions

This study provides the first confirmed record of *Hepatospora eriocheir* infection in Chinese mitten crabs from the Baltic Sea. Infection was detected in 9 of 15 examined *Eriocheir sinensis* and was associated with gross hepatopancreatic discolouration, tissue friability, spores in wet-mount preparations, histopathological lesions, and molecular confirmation by SSU rRNA sequencing.

The parasite sequences formed a single, well-supported clade closely related to published *H. eriocheir* sequences from the Thames Estuary and China. Host COI data revealed more than one mitochondrial haplotype among the examined crabs, indicating that infection was not restricted to a single maternal host lineage. In the context of previous studies on non-self-sustaining Polish mitten crab populations, the Baltic record is most consistent with host-mediated arrival through the wider North Sea-linked invasion system.

Further surveillance is required to determine whether *H. eriocheir* is sporadically introduced into the Baltic Sea, repeatedly imported from a limited number of donor populations, or more widely circulating across the North Sea to Baltic invasion corridor. Future studies should include broader geographic sampling, targeted testing of native and locally occurring decapod crustaceans, and higher-resolution characterization of Baltic *H. eriocheir* isolates using multilocus markers or genome-scale approaches, when suitable biological material becomes available.

## Figures and Tables

**Table 1:** Sample data for *Eriocheir sinensis* from the Vistula Lagoon, including sex, carapace width, colour of the hepatopancreas, molecular detection of *Hepatospora eriocheir*, and GenBank accession numbers.

Number of Individual	Sex	Carapace Width (mm)	COI GenBank Accession Number	Hepatopancreas Colour	Microsporidium PCR Result	Hepatospora GenBank Accession Number
1	male	74.8	ON815117	yellow	+	ON365845
2	male	73.0	ON815118	pinkish	+	ON365846
3	male	64.2	ON815119	yellow	−	-
4	female	73.5	ON815120	yellow/ orange	−	-
5	female	67.7	ON815121	yellow	+	ON365847
6	male	69.4	ON815122	yellow	+	ON365852
7	female	61.2	ON815123	whitish	+	ON365853
8	female	63.9	ON815124	yellow/ orange	−	-
9	male	49.9	ON815125	yellow	−	-
10	female	65.6	ON815126	whitish	+	ON365848
11	female	63.4	ON815127	yellow	−	-
12	male	60.7	ON815128	yellow/ orange	−	-
13	male	68.9	ON815129	yellow	+	ON365849
14	male	63.2	ON815130	whitish	+	ON365850
15	female	66.3	ON815131	whitish	+	ON365851

## Data Availability

The sequence data generated for this study are available in GenBank under accession numbers ON815117-ON815131 for *Eriocheir sinensis* COI and ON365845-ON365853 for *Hepatospora eriocheir* SSU rRNA.
